# Procalcitonin: A Powerful Rescuer on Surgical Floors

**DOI:** 10.7759/cureus.1446

**Published:** 2017-07-08

**Authors:** Ahsan Zil-E-Ali, Syeda Naqvi, Mariam Tariq

**Affiliations:** 1 General Surgery, Pak Red Crescent Hospital; 2 Jinnah Postgraduate Medical Centre, Jinnah Sindh Medical University (SMC); 3 Fmh College of Medicine, FMH College of Medicine

**Keywords:** surgery, necrotizing fasciitis, emergency, pro calcitonin, bacterial infection, trauma, prognosis

## Abstract

Necrotizing fasciitis (NF) is a rare but life-threatening medical and surgical emergency. It is characterized by necrosis of the soft tissue leaving the overlying tissue unaffected, which delays the diagnosis and treatment. Delay in recognition of the severity of necrotizing fasciitis might lead to serious morbidity and mortality. Diagnosis of NF relies on strong clinical judgment, predictable by severe pain, erythema, and a presence of air under the skin, but all of them are not always present. Management of NF is prompt surgical intervention and antimicrobial therapy. The effectiveness depends on the timely diagnosis of NF because it rapidly spreads and may cause irreversible damage.

Various investigations for necrotizing fasciitis have been proposed. However, misdiagnosis is not infrequent and more work is needed to identify the different presentations across the spectrum. We consider changing it to discuss the role of procalcitonin in the diagnosis of necrotizing fasciitis.There is scarce literature about its clinical role in necrotizing fasciitis although it has evolved not only as a prognostic marker but also as a way of differentiating between cellulitis and necrotizing fasciitis. It can also predict the future consequences of septic shock.

## Editorial

Necrotizing fasciitis (NF) is nearly always a fatal disease if not treated promptly in an adequate surgical environment and with appropriate medical treatment. The diagnostic scoring system for NF, laboratory risk indicator for necrotizing fasciitis (LRINEC), which was introduced in 2004, was mainly documented after trials on a brief cohort of 89 subjects and 225 controls [1]. Soon after the scoring system was introduced, the system became popular in clinical practice. But no health agency or standardizing organization has yet supported this scoring system, although we have come across multiple studies from different areas suggesting a true validity of this system.

But before we advocate it clinically, we have been warned to prospectively validate it in a global population and under different circumstances. It was suggested that LRINEC could be used for risk stratification and for better prognosis. But procalcitonin (PCT) is an equally important serology marker that is not a component of this scoring system, which raises questions for surgeons relying on LRINEC only.

Procalcitonin (PCT) is below the limit of detection in a homeostatic environment with as low as 0.01 µg/L in a clinical assay. The detectable higher levels in serum favor the bacterial inflammatory response and can theoretically be true for detecting NF. A score of ≥6 on LRINEC is considered NF but there are settings when other soft tissue infections (STIs) including cellulitis can have a ≥6 score, and the adjunct PCT value can help differentiate NF from STIs with as high efficacy as a 100% positive predictive value. The efficiency of PCT is not limited to differentiating NF from other STIs; rather, it successfully predicts the surgical eradication of infectious foci and is highly recommended one to two days postoperatively as an important clinical tool. It is amazing to see that PCT single-handedly served as a prognostic marker in not only just predicting mortality and chances of septic shock but also served parallel with sequential organ failure assessment score [2]. The meta-analysis of procalcitonin has also described that elevated levels in infectious diseases and NF assure the presence of sepsis and the following fatality. So it is encouraged that it be used with other investigations depending on the symptoms of the case. In the case of sepsis, inflammatory markers like lactate and C-reactive Protein (CRP) have been used to know the status of the condition; but still, it cannot be used as an initial marker for diagnosing sepsis. Especially, if we have high CRP levels, which are not very helpful in determining the severity of sepsis.

An interesting finding was that LRINEC scores with PCT had better predictability for severity and duration of hospital stay when compared to LRINEC score with CRP. The value of PCT levels after surgery for necrotizing soft tissue infections represents an important investigation to ensure eradication of the infection. While comparing PCT and CRP it was evident that PCT is a better marker of sepsis than CRP. The severity of sepsis-related organ failure was evaluated by the sepsis-related organ failure assessment score for PCT and CRP. The progression of PCT was found to have a closer correlation than that of CRP with the intensity of infection and organ dysfunction [3-4].

NF causes sepsis, severe sepsis, and other soft tissue infections. The fancied and practically easier LRINEC score should not make the surgeon overlook the importance of PCT [5]. Although PCT has been reported to be high in non-septic conditions like after liver transplantation, during severe and prolonged cardiogenic shock, in patients with heat shock, autoimmune conditions, severe pancreatitis, and rhabdomyolysis, its importance in diagnosing sepsis cannot be undermined. But the use of procalcitonin values with other inflammatory markers, clinical examinations, and imaging can help in diagnosing septic disease in early course, assist in differentiating different STIs and also measure the prognosis of the disease. With research, the validation of LRINEC can help it become a standardized system; but for now, PCT is as equally important in the clinical setting as any other score or serum value.
